# Integrating IoT and Blockchain for Ensuring Road Safety: An Unconventional Approach

**DOI:** 10.3390/s20113296

**Published:** 2020-06-10

**Authors:** Deepak Prashar, Nishant Jha, Sudan Jha, Gyanendra Prasad Joshi, Changho Seo

**Affiliations:** 1 Department of CSE, Lovely Professional University, Punjab 144411, India; deepak.prashar@lpu.co.in (D.P.); nishant.11702196@lpu.in (N.J.); sudhan.25850@lpu.co.in (S.J.); 2 Department of Computer Science and Engineering, Sejong University, Seoul 05006, Korea; 3 Department of Convergence Science, Kongju National University, Gongju 32588, Korea

**Keywords:** blockchain, hashgraph, IoT, OMNeT++, QoS, 5G technology

## Abstract

The Internet of things (IoT), the Internet of vehicles, and blockchain technology have become very popular these days because of their versatility. Road traffic, which is increasing day by day, is causing more and more deaths worldwide. The world needs a product that would reduce the number of road accidents. This paper suggests combining IoT and blockchain technology to mitigate road hazards. The new intelligent transportation system technologies and the subsequent emergence of 5G technologies will be a blessing, delivering the necessary speed to ensure both safety and quality of service (QoS). Hashgraph technology, a distributed ledger technology is used to create communication networks between the different vehicles and other relevant parameters. Scheduling the requests according to the priorities for ensuring better QoS quotient can be effectively done using hashgraph. We demonstrated how the hashgraph outstrips other equivalents platforms. The proposed model was simulated using OMNeT++ with proper design and network description files. A hardware implementation of the proposed model was also done. Messages were transferred between the vehicles and prioritized using a hashgraph. This paper proposes an effective model in reducing the accidents in terms of parameters like speed, security, stability, and fairness.

## 1. Introduction

The recent development of fifth-generation communication technology (5G) and Internet-of-Things (IoT) have ensured road safety specially in the field of vehicle maintenance, better transportation, navigation, and environmental controls. IoT equipped with a large number of sensors establishes communications with other vehicles and infrastructure devices and obtains most of its data. The recent work shows that the connectivity between various vehicular objects (humans, vehicles, sensors, etc.) enables real-time tracking of the flow velocity. 

Geolocation is one of the basic features to guarantee the road safety of the connected vehicles in which the connected vehicles are capable of interacting with each other. This has helped a lot in reducing accidents. Geolocation also provides a constant update of traffic conditions which enables frequent alerts, along with the traffic status, receiving updated information about the road conditions such as snow, potholes, etc.

Recently, a new integrable approach comprising of low power wide area network (LPWAN), blockchain protocols, and IoT have been the researcher’s choice. The combination of all these three have been named as IoT ecosystems. The so-called "smart cities" run seamlessly with this integrational technology; especially in traffic congestion in which the choking pollution and safety problems are major issues. Blockchain technologies have been better choices in resolving these issues because they have the potential to deliver cost-effective smart city solutions. It also reduces the cost of IoT communication effectively. The existing data transmission infrastructure over WiFi, Bluetooth, broadband networks, and long-term evolution (LTE) is highly limited. These legacy technologies were designed to link people and are not suited to move data at cost-effective rates. Internet-of-Vehicles (IoV) will serve as an integral vehicle-to-vehicle connectivity alternative to guarantee road safety. 

It also allows vehicles to respond to requests, including alarm signals such as “stop, crossing” and “speed limit,” or to emergency alerts, such as directions to the closest hospital in the case of a collision. This will help create smart roads that keep track of the number of vehicles that enter and exit. Hashgraph, a distributed ledger technology (DLT) based on blockchain technology, has recently emerged as a solution for the well-known problems of scalability and security.

Many works have been done on blockchain technology, but there remain many technical challenges that must be addressed to fully realize its potential. In this section, we discuss the challenges faced by blockchain technology and how we try to mitigate these challenges using hashgraphs in this work.

- Miners spend large amounts of time and resources validating and inserting the transactions into the blockchain.
- The ledgers of the blockchain are immutable, which means that data cannot be removed, changed, or transferred. Blockchain storage is very limited. It is possible to connect only a restricted number of transactions to blockchain.
- Due to storage issues, users pay a transaction fee to get their transactions included in the block by the miners. Larger transaction will cost a larger fee as it takes up more space in the block.
- Another limiting factor of blockchain is its low throughput and high latency. This is mainly due to slow transaction speed and minuscule amount of storage space and there is no appropriate ordering of transactions in blockchain.
- Blockchain’s consensus algorithm offers some limitations too. The consensus mechanism followed by blockchain is divided into two categories based on its type, i.e., Private or Public blockchain.
- Private blockchain is dependent on leader-based consensus mechanism which limits the usage only to trusted partners. However, the loosened security benchmarks make these systems potential targets for DDoS attacks.
- Public blockchain depends on consensus mechanisms such as ‘Proof of Stake’ and ’Proof of Work’. For these, every node must concur with the order of the transactions wherein they have taken place, which limits the number of applications where these technologies can be utilized.
- When two unique miners mine a block at the same time, the chain can part into two distinct forks. In this situation, the two forks will keep on approving blocks and include new blocks. When one of the chains approves a block before the other chain finishes it turns into the longest chain. The longest chain is the one with the longest most legitimate blocks joined and becomes the accepted chain. The transactions mined in the shorter chains are rejected. Rejection of shorter chains due to creation of forks does not guarantee that the transaction is irreversible.
- Creation of forks can cause selfish mining. This means miners can create a separate fork and hide newly generated blocks from the main blockchain and earn more.

Using hashgraph will improve quality of service (QoS) by making it a decentralized network where vehicles can communicate with or without third party involvement and assisted by 5G technology to exchange high-speed data between them. Therefore, IoV proves to be a thing of utility in daily life or a perfect example of realization of emerging technologies for practical needs ensuring sophisticated traffic management, proactive vehicles, and dynamic and diligent processing of requests. Further, opening possibilities for working with driverless cars too.

The main contributions of the paper are as follows.

1. To analyze the limitations of ensuring road safety using IoT and blockchain technology alone and to suggest a new approach for ensuring road safety by using hashgraph.
2. Integration of IoT with DLT through hashgraph is proposed and tested on the hardware.
3. The proposed approach is validated through simulations performed on OMNeT++.

The approach given in this paper is found more effective in resolving the limitations of the above-mentioned technologies in ensuring road safety which is discussed in detail in the succeeding sections. We simulated our approach in OMNeT++ and developed a hardware prototype for the same. 

Rest of the paper is structured as follows: Section 2 deals with the related work/background. Section 3 explains the significance of work. Section 4 deals with the system design and implementation. Section 5 deals with the simulations performed. Section 6 deals with the evaluation and analysis. The paper is concluded in Section 7.

## 2. Related Work/Background

Many researchers have done a lot of work in the field of vehicular ad-hoc networks (VANETs). However, there is very minimal integration of blockchain technology with IoT relating to VANETs. Still, some noteworthy work has been done. This section discusses those technologies and their related literature.

### 2.1. Interconnected Vehicles

Interconnected vehicles have access to Internet and can communicate through smart devices [1]. The data collection is done through integrated sensors and processing units which have been defined as On-Board Units (OBU) [2]. Many emerging technologies have been adopted by vehicle manufacturers [3] with the main aim to relieve drivers from the exhausting driving operations whilst allowing them to focus on other tasks. This will have a major long-term effect on urban planning and land use. The signal systems used in the traffic do not prove to be effective in terms of road safety. Moreover, autonomous vehicles use sensor systems to locate themselves along the route and interact with other vehicles and infrastructure [1]. While these developments look positive, problems are viewed by various researchers in many ways. Coppola et al. [1] identify privacy and data ownership as key concerns that need to be addressed in order to further improve and incorporate the technologies.

### 2.2. Privacy

Studies have shown that there are many risks that can be exploited if not protected, such as privacy breaching attacks that would jeopardize confidential user data [4]. The idea of privacy relates to ensuring that the information acquired is properly used, while third parties that process it should not extract intelligence from it. Although integrity and privacy are key issues surrounding connected vehicles, finding a balance between viable world solutions and security has proven to be a major challenge. Privacy is a key concern in the adoption of interconnected vehicles for both the private and public sectors [5,6]. 

Data misuse could precede targeted crimes, and the public sector could see the expansion and deployment of this technology blocked if the users consider it a threat. The data collected via connected vehicles and other ITS applications could potentially be useful for non-driver-related purposes. For example, the data may be used by state transportation departments or other road managers to evaluate patterns of road usage and to plan maintenance and improvements [7]. 

Although this is a major concern of the stakeholders involved, most of the current communication approaches do not consider aspects of privacy [8], all data is shared without reservations and without the owner’s permission, leaving them unprotected from attacker attempts to deanonymize users and link data pieces to understand their behavior. This attack is known as a linking attack. 

### 2.3. Data Ownership

In order to gain public recognition, the organization(s) handling sensitive data pledge to protect customer data; and past breaches have occurred either intentionally or unintentionally [9]. Moreover, in [8] the authors also focused on time-sensitive vehicles because a few minutes of data delay can lead to a disaster.

### 2.4. Blockchain Technology and Interconnected Vehicles

Information from several studies [7,10,11] indicates that blockchain can be an alternative to addressing the majority of the protection, privacy, and data ownership issues identified by taking advantage of the peer-to-peer structure of blockchain, lack of need for a trusted central authority, transparency, protection, anonymity, and privacy. 

Blockchain-based inter-vehicular communication architectures as proposed by Ali Dorri [8] is a good example. It elaborates how the convergence of interconnected vehicles and blockchain can solve information management security issues and open the door for further applications that sit on top of it. Their architecture consists of a semi-decentralized system consisting a layer of regional devices responsible for managing the blockchain, known as Overlay Blockchain Managers (OBMs). 

**Figure 1:**
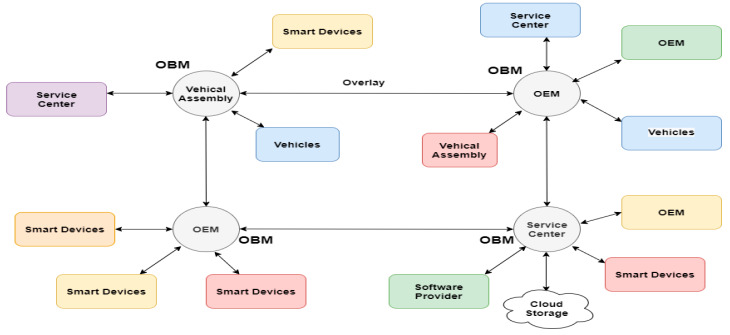
Blockchain based design of interconnected vehicles.

The vehicles communicate and exchange information with them, and the OBMs encapsulate the information and publicly distribute it through a shared ledger. The vehicles share their public keys with the OBMs, but they never reveal their real identity, enabling them to be anonymous, and the private information is stored in the on-board memory of the vehicles and exchanged only when the user wishes to share it, for example when they need to provide an emergency service to a particular location of the vehicles. The design is shown in Figure 1.

Ali Dorri [8] also suggested other applications that can benefit from their architecture, electric vehicles with smart charging services, insurance, and car sharing services, and are calling for more work on other applications, suggesting traffic management as a potential subject. 

There are few published studies on the subject of blockchain as a road traffic management enhancer. In his recent research, Akihiro Fujihara [12] investigated the collection of vehicle data through a blockchain network and identified an incentive-based system for encouraging the use of less congested roads and detecting car accidents and road conditions. His method uses PoW to mine the blocks in the network which, since it is based in Bitcoin, throttles the creation of a new block every 10 min on average. More recently, by opting for a new consensus method called Proof of Work at Proximity (PoWaP), Fujihara [13] has released further research improvements in this topic. Fujihara’s solutions [12,13] consist of beacons installed in roadside segments that sense vehicle proximity, get their address, and use it to estimate road conditions. These beacons can be contrasted with the OBMs in Ali Dorri [8] in the sense that they are both tools responsible for managing the blockchain and transmitting the relevant data, removing much of the vehicle’s burden and computational load. Similar work related to road safety using is presented in [14].

Xie et al. [15] developed a blockchain-based encryption system to enable vehicle IoT services, i.e., real-time cloud-based video monitoring and vehicle communication trust management. This research specifically demonstrates the 5G-VANET model with SDN-enabled and blockchain-based application scheduling procedures. Javaid et al. [16] provided confidence protection in VANETs by implementing a trustless network model using a blockchain and a certificate authority (CA) to sign IVs, as well as to revoke their registration where appropriate. Lu et al. [17] suggested a location-based services (LBS) dynamic key management scheme. The LBS session is broken down into various time slots with various session keys. A vehicle data authentication mechanism is subsequently described in [18], where the probabilistic verification technique is used to detect malicious behavior. In addition, the community signature with hash message authentication code (HMAC) is used in [19] to avoid the computation delay for the verification of the certificate revocation list (CRL). A trust-extended decentralized authentication mechanism (TEAM) for decentralized V2V communication is developed by Chuang et al. [20]. Notice that the structure for transitive trust relations is implemented to enhance authentication performance. Multiple authentication schemes have recently been developed [21,22] emphasizing lightweight VANET verification and preserving privacy. In particular, identity-based public key cryptography (IDPKC) [23] has been commonly used for the stable management of certificates in VANETs. Zhang et al. [24] initially suggested a batch signature check scheme for V2R communications. This scheme is nevertheless vulnerable to replay attack [25]. Meanwhile, Jung et al. [26] established a universal re-encryption scheme with a key establishment dependent on identity. Subsequently, the protection and repudiation authentication system (ACPN) for VANETs is presented [27]. Self-generated PKC dependent pseudo identities are implemented in their design. He et al. [28] subsequently established an efficient identity-based conditional privacy-preserving authentication (CPPA) scheme for VANETs. Additionally, virtual voting-based usage in block chain has been discussed [29]. Hussain et al. [30] proposed an e-mail-based model of social trust and trust based on social confidence in order to create and deal with the degree of trust knowledge. The key drawbacks of the information-powered trust models are inactivity and knowledge sparsity. Another concern is that vast quantities of information from various sources may contain repetitive data that could cause idleness or overshadow the noteworthy information. Shrestha et al. [31] proposed a new form of blockchain for use in VANET to resolve the issues of essential message propagation. They established a local ledger with communications about real-world events shared between the vehicles inside the country. They propose a decentralized blockchain that stores the trustworthiness of the node and trustworthiness of the message in a distributed ledger, ideal for safe distribution of the message. 

The studies undertaken so far have been focused on the collection of road data. However, much less work has been carried out in usage of those data for improving road traffic. Additionally, the privacy of those data has not been taken as a serious note. We tried to focus these issues, and eliminated these limitations using blockchain technology thus ensuring road safety. Further, our work uses hashgraph—a distributed ledger technology similar to blockchain for developing a system for ensuring road safety and is far better than blockchain technology and resolves the limitations of blockchain technology. Hashgraph and its comparison with blockchain technology is discussed in detail in Section 3.

## 3. Significance of Work

We suggest using hashgraph, integrating with IoT instead of using blockchain for ensuring road safety. Compared with the previous systems using blockchain technology and other methods for ensuring road safety, our approach guarantees that neither the data is stored at a single location nor is managed by the particular entity. We chose hashgraph algorithms because this algorithm does not require Proof-of-Work (POW) and provides minimal cost and high efficiency. This also does not require high power and electrical supply for computation. 

**Figure 2:**
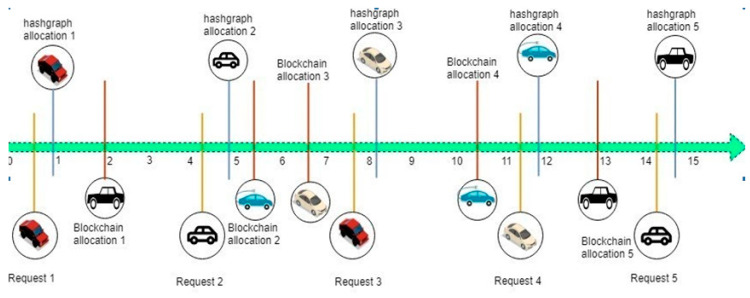
Transaction-based comparison between a hashgraph and a blockchain with respect to time.

Different blockchain platforms possess different speeds (transaction/speed). For example, bitcoin has a speed of 6–10 transactions/second, Ethereum has 14–20 transactions/second whereas Hyperledger Sawtooth can perform thousands of transactions/second. However, our proposed solution gives hundreds of thousands of transactions/second with simpler and efficient processes guaranteeing the road safety. The proposed work has the flexibility of choosing the order of speed of transactions, postponing them, or even stopping them from reaching the block. However, a timestamp consensus is used in hashgraph which prevents individuals from changing the order of transactions, thereby solving the privacy and security issues faced by IoT and blockchain. Figure 2 shows how a hashgraph is more effective than a blockchain, because the timestamp ensures that the proper action is taken at the right time without any serializability issues.

Our approach also ensures network security by various techniques, including cryptographic encryption, Byzantine fault tolerance (BFT), ACID enforcement, and tolerance to Distributed Denial of Service (DDoS). This hashgraph feature makes it more secure than a blockchain. Using a hashgraph can boost quality of service (QoS), creating an open network where vehicles connect with or without third-party intervention, and using 5G technologies to share high-speed data between them. Furthermore, the data in the blockchain are not immutable. Blockchain scalability continues to be an issue. Due to storage problems, users must pay a transaction fee to get the miners to include their transactions in the stack. Larger transactions may be expensive to include. The chain can divide into two distinct forks when two unique miners mine a block at the same time. The two forks will both proceed to accept blocks in this case and will include new blocks. 

The first chain to accept a block becomes the longest chain and is followed by the largest legal block, becoming the chain that is acknowledged. In the shorter chains, the transactions extracted are refused. Performance, reliability, and security are the pre-eminent variables that make our approach a remarkable innovation, fit for contending with blockchain and other methods. IoV is a perfect example of how emerging technologies can be applied for practical needs, such as advanced traffic management, responsive cars, and diverse and diligent request processing. The technology opens up even more opportunities for operating driverless cars. This renders the hashgraph a promising function to use in vehicles, greatly reducing accidents [32].

The technology behind the hashgraph is extremely fascinating, but its real potential and efficiency will only be understood once it is made available to the public and non-permission-based networks.

## 4. System Design and Implementation

**Figure 3:**
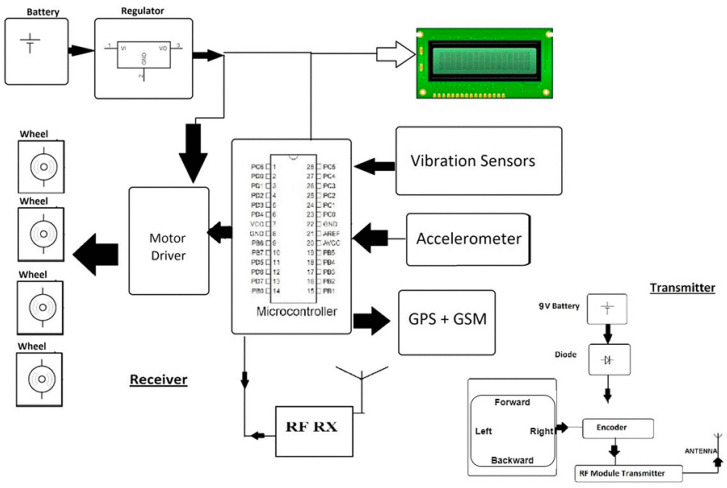
Circuit diagram of the proposed hardware for road safety.

This paper proposes road safety measures based on the hashgraph integrated with the IoT system, which can easily be implemented. Specifically, we propose hardware implementation of an accident avoidance system through the Global Positioning System (GPS) notification, and its implementation with a hashgraph. The planned hardware circuit diagram is depicted in Figure 3. 

When a motorist experiences an accident leading to injury or damage, there may be nobody around to help. In such a situation, the system acts as an accident prevention and recognition mechanism that assembles the data and transfers it to the nearest person or to someone listed by the driver as an emergency contact. A GPS receiver and a Global Switch Mobile (GSM) module are also used in the system. The GPS receiver is used to identify the vehicle location, while the GSM module is used to submit an optimized SMS with coordinates and a Google Maps link. In a vehicle warning program, an accelerometer can be used to detect dangerous driving. It can still be used after a collision to identify the car crash or rollover. A severe accident can be identified through accelerometer signals. As soon as a vehicle experiences an accident, it would be detected by the vibration sensor. If a vehicle flips over, the microcontroller would illuminate, imparting this change of location to a GSM module. Then the GSM module assistant would communicate the details of the accident through the Google Maps app. The individual can then be monitored from anywhere in the region through GPS. 

The next step is to integrate the hardware with the hashgraph. To track the operations a hashgraph uses direct acyclic graphs. One may assume that every node in the network has its own graph, and that graph is transmitted to every other node. The same thing is done in blockchain technology, using a chain. It is achieved using a formal approach such as a gossip protocol, virtual voting, etc. Maintaining honest agreement in the network provides protection to attacks. However, one needs to understand the processes behind the hashgraph method to understand how the proposed integration will operate. Figure 3 shows the block diagram of the hardware developed. 

### 4.1. Working of Hashgraph

**Figure 4:**
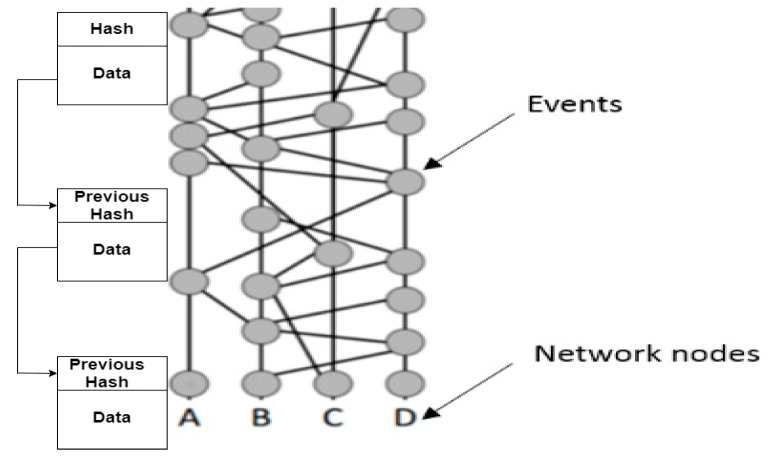
Diagrammatical difference between blockchain and hashgraph.

The data is stored in the form of events in hashgraph containing transactions and a timestamp similar to blocks with two regular hashes. Since a hashgraph exploits directed acyclic graph (DAG) to record the operations, increasing node in the network can have its own graph and that graph is with every other node. In blockchain technology the same thing is achieved using chains. This happens using a comprehensive process. This mechanism leads to various ideas like a gossip protocol, virtual voting, etc. Holding an honest consensus within the network guarantees resistance from attacks. Figure 4 shows the difference between a blockchain and hashgraph.

**Figure 5:**
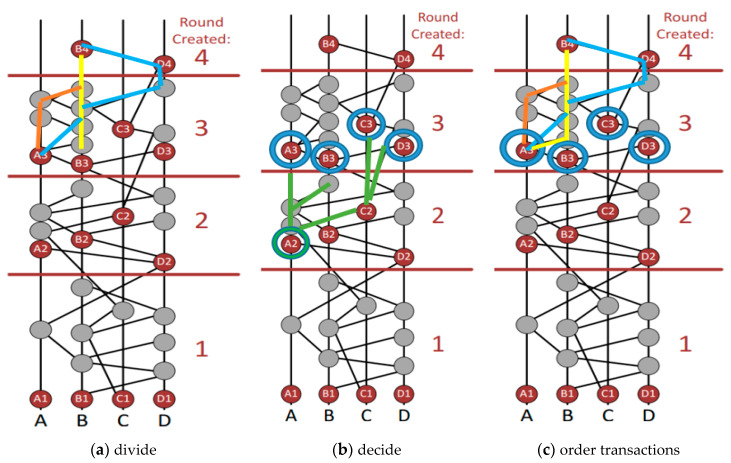
Round creation in hashgraph.

- *Gossip Protocol:* Gossip protocol requires the fast exchange of information between the nodes. Passing from one node to another, an incident can be communicated through the entire network. Every event needs to reach every node. The network, not the sender, is responsible for disseminating the details. This constant information sharing gradually builds the hashgraph, which continues to grow.
  It is interesting to note that there is no actual graph stored in the memory. It is more of a concept. Incidents are stored in memory, however. The hashgraph data are secured cryptographically. The network formed accommodates the communication history of the process. Furthermore, it is not enough to ensure that all the events know about each other. It is equally necessary that events agree on a certain linear order of transactions that are recorded inside the events.
  It might appear that once gossiping begins, every event becomes aware of every other event and hence consensus is reached, but this is not true. There may be moments when some recent events are yet to be communicated, and this leads to virtual voting. Both in blockchain and in hashgraph, the gossip protocol is used to distribute the information through the network. Basically, every time a node is sent it communicates a new transaction to its neighboring nodes, and so on.
- *Proof-of-Work Consensus Protocol:* This protocol is used by most of the renowned blockchain technologies like Bitcoin and Ethereum. The nodes are trying to compete to acquire the authority to modify their data to the blockchain on a quite complex mathematical challenge. Validating a block takes an average of 10 min and consumes a ludicrous amount of energy.
  Throughout the protocol, Bitcoin and Ethereum nodes run a protection algorithm that verifies all transactions to ensure that they do not allow a fraudulent transaction. Even if the node wants to manipulate and add a block with a double spend transaction, the next node to add a block to the blockchain would reject it, communicate with the node before it, and thus delete the fraudulent block transactions. Mining costs are expected to dissuade miners from attempting these sorts of attacks on a blockchain, but if many nodes are deceptive, the duration of the “fraud chain” can increase. This means that the consensus is never necessarily reached in the Work Consensus Proof, but just attempted, which also explains why a transaction receiver usually waits for verifying several nodes to verify a transaction. In a hashgraph the consensus protocol is based on the link graph and the network nodes do not require any additional effort. This process is described below in virtual voting.
- *Virtual Voting and Round Creation:* The transactions, consensus, and nodes are some of the main terms for understanding virtual voting. Virtual voting is somewhat similar to democratic voting. It determines a witness’s vote based on witnesses or events. In technology such as Bitcoin, a block makes consensus decisions if it is fast enough to be the first to solve the hash, but in virtual voting, the decision node is selected through a complicated process consisting of three stages: (a) divide, (b) decide, and (c) order transactions. The process of round creation is shown in Figure 5. Here, The Hashgraph is broken into rounds. Each time an event can connect more than 2/3 of the first events of the current round by more paths than 2/3 of the node population is created a round. In doing so one event sees the other event strongly. A3 is strongly seen in Figure 5 (Population of node = 4, Path Number Connection = 3). Each node of the newly created round must acknowledge whether it contains the data of the previous node or not. Here previous node refers to the node of the previous preceding round. Verification is done once this acknowledgment is received. In this figure all first nodes in the third round must have decided on the data in A2. The final stage is to collect answers from the third-round nodes. The fourth-round nodes are required to do so. They need to see the third-round node very strongly. When one of the fourth-round nodes succeeds in gathering a super majority (more than 2/3 of the population) of positive votes over the data in the second-round then this data is found to be consensus.
  - Divide Round: Rounds are generated on the basis of events which are clearly seen. If A can see 2/3rd round *t* events even, then round *t*+1 is allocated. All the first events are called witnesses in a round, and only the witness events are allowed to send or receive virtual ballots. In the figure, all witnesses are A1, B1, C1, D1, A2, B2, C2, D2, A3, B3, etc., as they are the first events of rounds 1, 2, 3, respectively, as shown in Figure 5. This process takes place in Decide Fame. Algorithm 1 shows the creation of divide round [33].
**Algorithm 1: Divide Round**1: **Process** Div Round2: **For** Every Event A3:  M ← Maximum Parent Round for A4: **Else**5:  M ← 1 *// no Maximum Parent Round exist*6:  **If** A > 2t/3 M *// A sees more than 2t/3 rounds than M witnesses*7: A.round ← M+18:  **Else**9:   A.round ← M10:  A.Witnesses ← no self-parent of A exists11:  Or (A.round > A.self-parent.round)
  - Decide Fame: Whether an event (witness) is famous or not, is determined in this round. You can call a witness famous because other next-round witnesses will see it. For example, B2 is seen by A3 via B3, it is also seen by C3 through D3 and B3, hence it is seen by four next-round witnesses namely A3, B3, C3, D3 (Round-3 witnesses). B2 is popular for this. This exercise takes place for all witness cases. When two-thirds of a population (referring to stake) sees an incident then a super-majority is said to see it, meaning that it is strongly seen. Vote counting is achieved by events involving witnesses. From B4, A3 can be seen strongly through paths via A, B, and D, which is a super-majority. B4 can also see B3 and C3 from A, B, and D strongly, too. It is also able to see D3 strongly, through A, B, C, and D. This confirms B2 is undeniably successful after the counting of votes. Algorithm 2 shows the creation of decide fame.
**Algorithm 2: Decide Fame**1: **Process** DecFam2: **For** every event A ordering from earlier to later on basis of rounds round3: **If** A.Witnesses && B.Witnesses && B.round > A.round4: **Else**5:        P ← B.round – A.round6:  Q ← Witness set events in round7: B.round −1 *// B can see strongly*8: R ← Q9: containing majority Votes (‘TRUE’ in case of a tie)10:  F ← Q events with R votes11: **If** P = 112:  B. Votes13: **Else**14: **If** P mod J > 0 // *for normal rounds*15: **If** P mod J > 0 // *for normal rounds*16: **If** F > 2*t/3 // *take decision when in supermajority*17:  A. Fam← R18:  B. Votes ← R19: **Break**;20: **Else** // *only Votes*21:  B. Votes ← R22: **Else** // *Coin Round*23: **If** S > 2*t/3 // *vote if it is a supermajority*24:  B. vote ←   R25: **Else** // *Coin flipping occurs*26:  B.Votes ← mid bit of B.sign
  - Order of speed of transactions: After a consensus has been reached on the fame of certain events, another consensus can be achieved regarding the order of transactions. This is done by sorting out timestamps and consensus on older events. To understand this step, the concept of “received round” is key. An event E has a received round Y if Y is the first round where all the unique famous witnesses are descendants of E. For example: E has a received round Y. Then a new unique famous witness W is created by A in round Y. However, W has a self-ancestor V, which has the knowledge of E beforehand. Additionally, when it is first created, it is given the timestamp S. Therefore, S is the time when A first learned of E. Therefore, the received time of E can be calculated by taking the median of all such timestamps created by multiple members in round Y. In this way consensus order is reached. All events are sorted according to their received rounds. If two events have the same rounds, they can be sorted by received times. If they are still tied, they are sorted by their signatures. Events are split by sorting with respect to signatures after the XORing is performed on these signatures with other unique famous witness signatures in the received round [34].
**Algorithm 3: Order Finding**1: **Process** FindOrd2: for every event A3:  if non-existence of event B for round M in or before round M having B.4:  witnesses = ‘TRUE’ && B. fam = UNDECIDE && A ϵ ancestor of every round with unique fam witnesses && is ‘FALSE’ of any round before round5: **then**6: A. roundReceive ← M7: Q ← every event set W so that V ϵ self-ancestor of round unique fam witnesses, && A ϵ ancestor of W not belongs to self-parent of W.8: consenTime←median of //*Consensus Timings Timestamps*9:   **for** all events in Q10: **return** each and every event having roundRecieve not ‘UNDECIDE’, sorted by roundReceive, && ties sorted by consenTime, through white sign

### 4.2. Requesting of Message Ordering 

As explained in the preceding section, the ascending order of events is generated using the hashgraph consensus timestamp. However, a necessary priority set of messages and also a priority set based on the time-limit of messages can be set. The ordering of timestamps will not always be the same as the ordering based on the priority of the notification requests the deadline received by applying for vehicles. It is assumed that the final sequence of events will include a three orders-hashgraph timestamp, priority sequence, and deadline of the requested messages or flags. Therefore, it is appropriate to receive a final order after taking all three sets of event-orders into consideration. 

Let us consider that there are *X* number of events present in the network. Let *V1*, *V2*, and *V3* be the transition-order vectors generated by vehicle-nodes through hashgraph consensus algorithm, expected priority order, and least deadline time, respectively. Let *G1*, *G2*, and *G3* be the weights associated with the consensus order of hashgraph, pre-defined priority-order and least deadline time order, such that *G1 + G2 + G3 = 1.* The values in vectors *V1*, *V2*, and *V3* are multiplied with the respective weights *G1*, *G2*, and *G3* and the results are stored in vectors *Y1*, *Y2*, and *Y3*, respectively.

*Y*1(*k*)_*k*=1_ = *G*1(*V*1(*k*)_*k*=1_) (1)

*Y*2(*n*)_*n*=1_ = *G*2(*V*2(*n*)_*n*=1_) (2)

*Y*3(*l*)_*l*=1_ = *G*3(*V*3(*l*)_*l*=1_) (3)

The events corresponding to the three vectors *V1*, *V2*, and *V3* are the same. The values in vector *V1* are compared to values in vector *V2*. If the first value in vector *V1* is smaller than all values in vector *V2* then that particular event in both the vectors *V1* and *V2* are eliminated and the event is stored in another vector *F*. Otherwise, if any value in vector *V2* is found smaller than the first value in vector *V1*, then that particular event is eliminated and the event is stored in another vector *F.* The process is repeated until all the events in vector *V1* and *V2* are eliminated and vector *F* gives the final ordering of the offloading tasks considering both the hashgraph consensus timestamp and pre-defined priority order. The new weight for vector *F* being the average of *G1* and *G2*, denoted as *G_F_*. Now, the new vector *F* is multiplied by weight *G_F_* and we obtain vector *D*, as shown in Equation (4).

*D*(*P*)_*P*=1_ = *G_F_* (^*m*^*F*(*P*)_*P*=1_) (4)

where, *G_F_* = (*G*_1_ + *G*_2_)/2. 

Now the same process of elimination is carried out on vectors *D* and *V3.* That is, vector *D* and vector *V3*, which was obtained by multiplying the deadline order with weight *G3* to finally give the required order vector *H*.

## 5. Simulation

**Figure 6:**
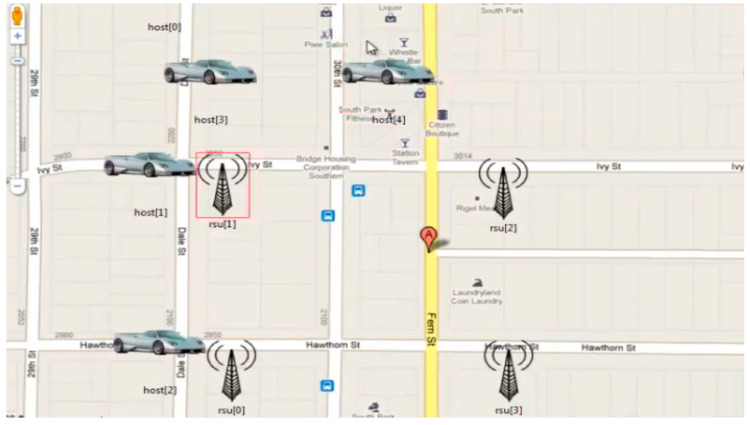
Simulation of suggested solution.

**Figure 7:**
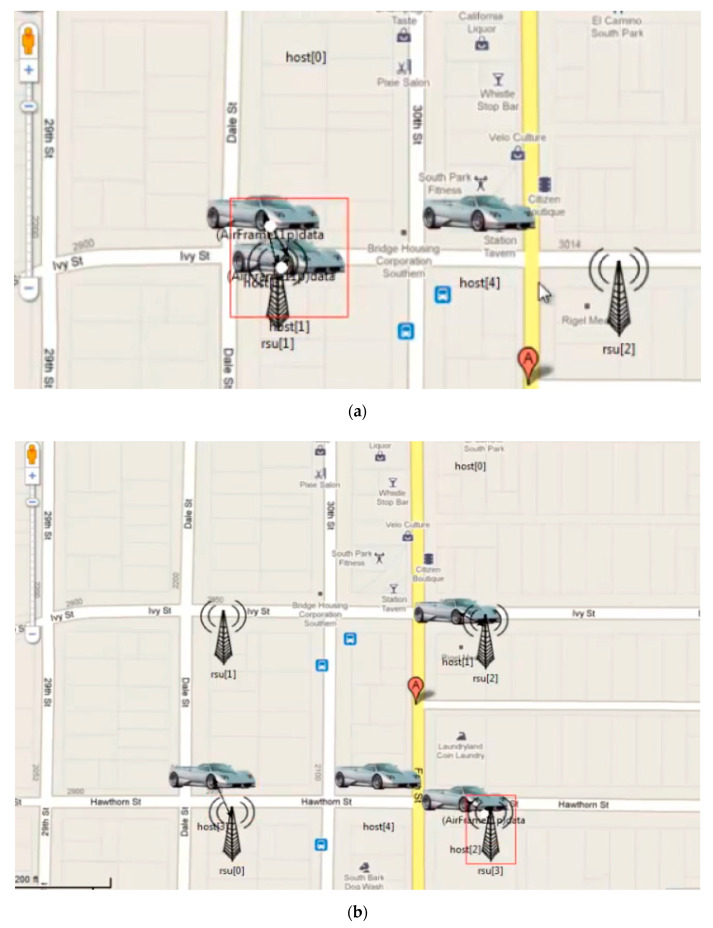
(**a**) Bandwidth allocation ongoing between the vehicles. (**b**). Bandwidth allocation and message passing between vehicles.

The proposed model based on the hashgraph was validated through a simulation on OMNeT++, as well as by fabricating a piece of IoT hardware to synchronize the model during the deployment. OMNeT++ provides a network simulation facility in the form of a modular extensible component-based C++ library. Figure 6 shows the simulation of the proposed solution followed by the bandwidth allocation and message passing between the vehicles as depicted by Figure 7a and Figure 7b, respectively.

**Figure 8:**
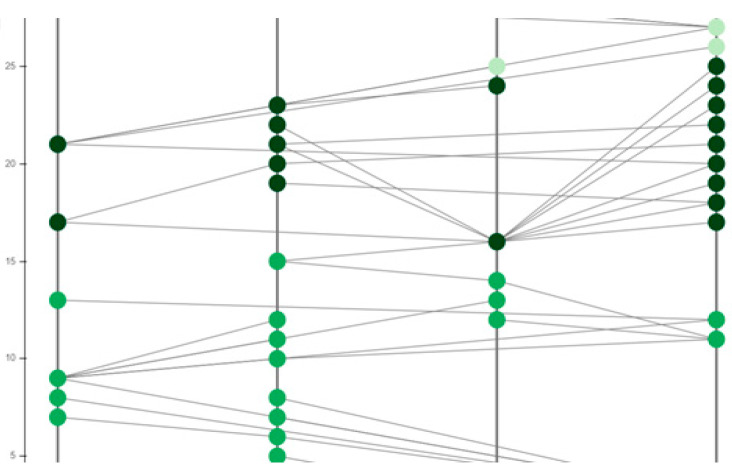
Implementation of hashgraphs on OMNeT++.

The proposed model based on the hashgraph was validated through a simulation on OMNeT++, as well as by fabricating a piece of IoT hardware to synchronize the model during the deployment. OMNeT++ provides a network simulation facility in the form of a modular extensible component-based C++ library. Presented are the results of a basic simulation executed on OMNeT++ pertaining to the gossip protocol of the hashgraph running between the vehicles as shown in Figure 8. The messages transferred are of four types: security messages, help messages, event messages, and multimedia messages. OMNeT++ has an excellent graphic user interface. The components of the simulation, such as initialization, handling, and forwarding of messages, are written in C++. These components, on which the design part of the simulation is focused, are assembled using network definition files written in the NED language. Some of the advantages of using OMNeT++ rather than other simulators are that it is well structured, that the source code is publicly available, and that it is not limited to network protocol simulation. Its hierarchical nature helps in large-scale simulations, making components reusable. It helps in analyzing results as well. The vehicles which are to be connected are represented as nodes in the simulator. Multiple nodes are connected to each other, as shown in Figure 8 with lines representing the connections. In a blockchain, message requests made by the nodes travel through the network in random order; however, when event order is obtained with a hashgraph, messages travel in the order of the timestamps produced by hashgraph. In this case, the vehicles represented by the nodes in the simulation are actually events in the hashgraph, and the messages which travel in the network are transactions taking place in hashgraph. Additionally, the codes can be fixed so as to share messages according to various event orders, such as the predefined priority order or the deadline-based order. The nodes decide on their own and regularly forward transactions, state and status information for roadside units (RSU) or other entities. Those stable components also have a direct relation to the Internet Vehicles which do not have access to roadside units, and can also use a mesh-based approach where an ad hoc network relays their data to the destination.

## 6. Comparative Analysis

As discussed in the preceding sections, our work is based purely on the following parameters. They are transaction speed, stability, security, and fairness. These are the factors which were not considered in any of the previous works. The detailed analysis of these parameters with respect to our work are as depicted in Table 1.

1. Transaction Speed:
  - Bitcoin and Ethereum are restricted by their consensus protocol to 6 and 14 tps, respectively, while the hashgraph based approach given by us is limited only by the internet bandwidth to 260k tps which is approximately five times greater than the visa network.
  - The consensus algorithm of hashgraph can process thousands of transactions per second. Consensus latency is very low while it provides very high throughput. Hashgraph technology is quick as far as utilizing the gossip protocol which spreads messages between network users. These messages are selectively optimized to diminish the communication overhead.
  - The identities of all nodes are known beforehand in permissioned distributed ledgers and the network is not open to the arbitrary participants. The prior knowledge of the participating nodes’ identities provides a strong protection against Sybil threats and facilitates consensus building. This means that no framework of Sybil protection needs to be placed in between and thus the performance can be significantly improved when compared to the public blockchain.
  - Since hashgraph is currently a private distributed ledger, its output is compared with other private blockchains, such as IBM HyperLedger Fabric (700–800 transactions per second) or Red Belly (400,000–500,000 transactions per second). Its performance must not be collated with public blockchains such as Bitcoin or Ethereum (10 transactions per second), as their speed is low. Hashgraph has yet to provide specific technical information to deploy as a public ledger for its implementation.
2. Fairness: In blockchain a node decides in which order a transaction should be kept in a block. However, this may be viewed by end-users unethically and prevents the building blockchain based applications like share market. In hashgraph, the timing protocol is absolutely fair to all users. Since our method is based on the gossip protocol, which implies that if a node randomly selects its successors homogeneously, there is some likelihood (e.g., one-third), if neighbors of the node are randomly selected uniformly that all the nodes selected will be Byzantine or dishonest. These dishonest successors will avoid the transaction from being transferred to the next group of nodes, thereby preventing the transaction from hitting two-thirds of the network which would end in an unacceptable result for the fair creator.
3. Security: Asynchronous Byzantine fault tolerance (A-BFT) is the special distributed consensus technology which is used by hashgraph. This ensures that the protocol will always reach consensus with the only assumption (i.e., less than 1/3 of the nodes are malicious). It is proved that the incompressible limit for a system to be BFT is 1/3 of nodes. In addition, the hashgraph is the only decentralized protocol that is ‘bank-grade level of security’ and can work with various institutions.
4. Stability: Our proposed system is stabile as it ensures security because the possibility of forking is minimized by allowing advanced legitimate controls. Technical controls include signed state proofs, ledger ID, and even forks are also taken care of in this way.

**Figure 9:**
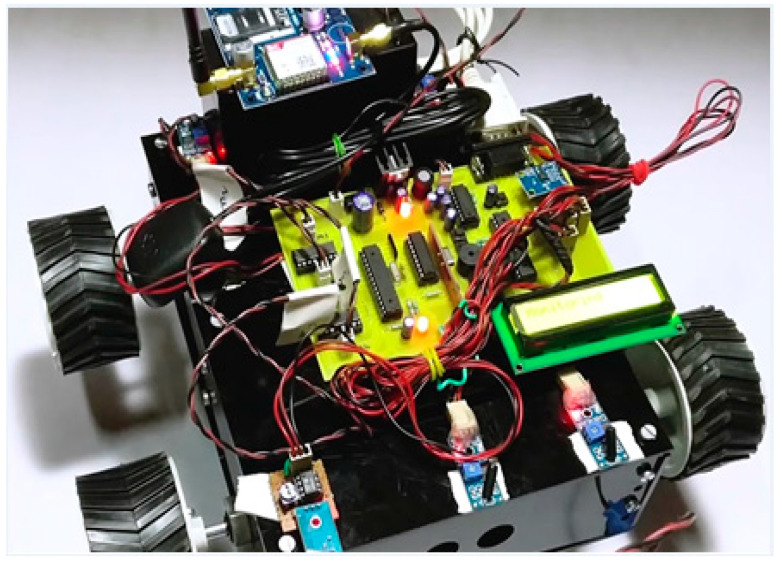
Fabricated hardware for a vehicle for integration with the hashgraph.

From the parameters discussed above, our approach is more efficient and robust in solving the challenges faced by blockchain and other technologies and can be used for developing the systems for ensuring road safety. The image of the hardware prototype developed is shown in Figure 9.

This fabricated hardware for a vehicle for integration with the hashgraph contains Atmega microcontroller, crystal oscillator, accelerometer, vibration sensor, regulator, RF transceiver module, robotic chassis, an LCD display, GPS receiver, global system for mobile communications (GSM) transceiver, DC motor, transformer, and other circuitries. The programs are written in Arduino and C computer programming languages. The vehicle has to verify its identity and join the testnet for the integration with hashgraph. Before joining the network, a unique access code is required to be verified by the vehicle or its owner to avoid any tampering with the network. This will generate a public and private key that will enable the model to join in the main network of all the vehicles. The proposed model is more efficient and powerful than existing methods in terms of transaction speed, stability, security, and fairness, as described in Table 1.

## 7. Conclusions

The admonitions to drive safely to avoid accidents are ignored by large segments of the population, and road accidents are becoming more frequent day by day. The advent of technologies like IoT and IoV has reduced accidents somewhat, as the vehicles are now equipped with relevant technology, but accidents are still too frequent. The technique proposed in this paper is an efficient model for ensuring road safety. A smart vehicle running on a smart road could respond to many different requests, from reporting an accident ahead to downloading a song at the blink of an eye. Research in this area is ongoing, and the integration of blockchain technology can prove quite effective in this area. The use of hashgraphs clearly makes the system more reliable and stable. Moreover, integrating the hashgraph with the IoT-based hardware developed during the course of this research has proven even more effective in preventing road accidents. Authentication and user revocation in the VANET are two essential aspects of protection. It is highly critical that these tasks are carried out promptly and effectively. The past works addressing these issues are deficient in minimizing the dependence on the trusted centralized authority and therefore do not provide dispersed and decentralized protection. This paper proposes a DLT (hashgraph)-based authentication and revocation system for vehicle networks, not only reducing overhead computing and communication by minimizing reliance on a trusted identity verification authority, but also speedily updating the status of relocated vehicles in the public blockchain ledger. The proposed methodology makes the roadside units verify a vehicle’s identity on the lane. The method’s efficiency and consistency were validated and analyzed on various parameters such as, security, fairness, stability, and transaction speed. The proposed decentralized framework will mitigate delay in reactions through the innate agreement process that is the USP of the hashgraph. In the future, we will do more experiments and present the results.

## Figures and Tables

**Table 1:** Comparative analysis between our proposed work with previously published work.

Parameters in the Proposed Method	Objectives of the Parameters in Proposed Work	Existing Works in Which These Parameters are Used	Objectives of the Parameters in Existing Works
Transaction Speed	The hashgraph based approach in our method is limited only by the internet bandwidth to 260k tps which is approximately five times greater than the visa network.	Xie et al. [15]	Bitcoin and Ethereum are restricted by their consensus protocol to 6 and 14 tps, respectively
Stability	Possibility of forking is minimized by allowing advanced legitimate controls	Zhang et al. [24]	No legitimate controls Suggested a batch signature check scheme for V2R communications. However, this scheme was vulnerable to replay attack.
Security	Our method proves that the incompressible limit for a system to be BFT is 1/3 of nodes which is better than any other proposed method	Lu et al. [17]	Suggested a location-based services (LBS), with dynamic key management scheme. No quantitative analysis has been done regarding security to date.
Fairness	All the nodes selected are either Byzantine or dishonest.	He et al. [28]	Not necessary. An efficient identity-based conditional privacy-preserving authentication (CPPA) scheme established. Worked specially in VANETs.
